# Surgical Management of Pelvic Floor Disorders and the Utility of Preoperative Labs

**DOI:** 10.1007/s00192-025-06307-7

**Published:** 2025-10-01

**Authors:** Tara Samsel, Sarah Ashmore, Jinxuan Shi, Kimberly Kenton, Margaret Mueller

**Affiliations:** Section of Urogynecology and Reconstructive Pelvic Surgery, University of Chicago, Chicago, IL USA

**Keywords:** Pelvic floor disorders, Preoperative laboratory testing utility, Preoperative testing, Preoperative testing guidelines

## Abstract

**Introduction and Hypothesis:**

Current national guidelines advise against routine preoperative laboratory testing to assess risk of perioperative events prior to surgery for pelvic floor disorders (PFDs), yet many women undergo unnecessary testing. The primary objective was to determine the clinical utility of preoperative laboratory assessment prior to surgical management of PFDs. Secondary objectives include rates of case cancellation or postponement owing to laboratory abnormalities and factors associated with testing.

**Methods:**

A retrospective study was performed of all surgeries within the Division of Urogynecology and Reconstructive Pelvic Surgery at a single academic medical center between 1 January 2021 and 31 December 2024. Patient demographics, clinical characteristics, and preoperative laboratory assessments were abstracted from the medical record. Case cancellation or postponement was documented. Group comparisons were performed using Student’s *t* test and Chi-squared and Fisher’s exact test for continuous and categorical variables.

**Results:**

During the study period, 634 surgeries were performed for PFDs. Four hundred sixty-nine (74%) women had preoperative labs performed. Laboratory abnormalities were rare and did not result in any changes to surgical management. Patients with diabetes mellitus were more likely to have preoperative labs (*p* = 0.02); otherwise, medical co-morbidities were similar in the two cohorts. Patients who had preoperative labs were more likely to have a hysterectomy (*p* < 0.001), sacrocolpopexy (*p* = 0.004), uterosacral ligament suspension (*p* = 0.001), anterior repair (*p* < 0.001), and posterior repair (*p* < 0.001).

**Conclusions:**

Despite the majority of patients undergoing preoperative laboratory testing, clinically meaningful abnormalities were rare and did not change surgical management. Policies surrounding preoperative testing should be re-evaluated to reflect current national guidelines.

## Introduction

Women undergoing surgery for pelvic floor disorders are generally healthy, and the procedures are predominantly low risk and elective. The risk of perioperative transfusion following reconstructive pelvic surgery is as low as 1% and women undergoing surgery for pelvic floor disorders often have low rates of significant medical co-morbidities [1–3]. The elective nature of the surgical procedures allows time for optimization of medical co-morbidities to decrease the risk of perioperative complications.

Preoperative laboratory testing is performed ahead of surgery to assess the risk of adverse perioperative outcomes. Current American Society of Anesthesiology (ASA) and National Institute for Health and Care Excellence recommend against routine testing and instead recommend selective testing based on patient comorbidities or invasiveness of the procedure [4]. Despite these recommendations, women often undergo preoperative laboratory testing ahead of surgery for pelvic floor disorders, despite their known low utility [3, 5]. Hospital policies, including those at this institution and consistent with others in the literature [3], are lagging behind current national recommendations, and thus providers continue to perform routine preoperative laboratory assessment, which is associated with low patient satisfaction, increased laboratory costs, and inappropriate waste of laboratory resources and time [6, 7].

Given the current national guidelines and low-risk population of women undergoing surgery for pelvic floor disorders, we performed a study with the aim of evaluating the utility of preoperative laboratory testing. Specifically, we aimed to examine the rate of clinically meaningful laboratory abnormalities identified on routine preoperative labs and their impact on the surgical plan.

## Materials and Methods

A retrospective cohort study was performed of all surgeries for pelvic floor disorders within the Division of Urogynecology and Reconstructive Pelvic Surgery at a single academic medical center between 1 January 2021 and 31 December 2024. Institutional Review Board (IRB24-0704) approval was obtained prior to study performance. Surgeries performed for any pelvic floor disorder (i.e., urinary incontinence, fecal incontinence, pelvic organ prolapse) were included. Charts were reviewed to determine if preoperative laboratory assessments were completed ahead of surgery. Preoperative labs included type and screen (T&S), complete blood count (CBC), and basic or complete metabolic panel (BMP or CMP). The primary outcome was the rate of clinically meaningful laboratory abnormalities detected prior to surgery. Hemoglobin (Hgb) <10 mg/dl, platelet count (Plt) <100 × 10^3^/ml, creatinine (Cr) >1.0 mg/dl, sodium (Na) <135 mEq/l, or >145 mEq/l, potassium (K) <3.6 mEq/l or >5.1 mEq/l, and/or glucose >200 mg/ml were defined a priori as clinically meaningful laboratory abnormalities based on clinical opinion. Rate of case cancellation or postponement due to laboratory abnormalities and factors associated with the obtainment of preoperative labs were assessed as secondary outcomes.

Patient demographics, clinical characteristics, preoperative laboratory assessments, and surgical details were abstracted from the medical record. Surgical cases were categorized into major and minor procedures. Hysterectomy, apical suspension, and colpocleisis were included as major procedures. Minor procedures included midurethral sling, sacral neuromodulation, intravesical Botox, isolated anterior and/or posterior repair, and cystoscopy.

Statistical analysis was performed using IBM SPSS Statistics version 29 (SPSS, Armonk, NY, USA). Standard group comparisons were performed. Categorical variables were compared using Chi-squared and Fisher’s exact tests. Independent Student’s *t* test or Mann–Whitney *U* test was utilized to assess continuous variables depending on data distributions.

## Results

A total of 634 surgeries were performed for pelvic floor disorders during the 4-year study period. Table 1 displays demographics, clinical characteristics, and surgical details for the entire cohort. Mean (SD) age of the entire study cohort was 54.5 (±16.3) years, with a mean body mass index of 26.8 kg/m^2^ (± 10.4). White race was most common (49%) and the majority of women had private insurance (51%), with ASA class 2 physical status (62%). Three hundred and ninety four surgeries (61%) were considered major procedures, whereas 240 (38%) were minor procedures. Table 1 includes a breakdown of surgeries performed.

**Table 1:** Patient demographics, clinical characteristics, and preoperative laboratory testing

Variable	Entire cohort (*N* = 634)	Labs (*N* = 469)	No labs (*N* = 165)	*p*
Age (years)	54.5 (± 16.3)	54.6 (± 16.6)	54.3 (± 15.4)	0.18
BMI (kg/m^2^)	26.8 (± 10.4)	27.1 (± 10.6)	26.5 (± 9.9)	0.62
Race				0.004
American Indian/Alaska Native	0 (0%)	0 (0%)	0 (0%)	
Asian/Indian	15 (2%)	9 (2%)	6 (4%)	
Native Hawaiian/Pacific	2 (0%)	2 (0%)	0 (0%)	
Islander				
Black/African American	198 (31%)	165 (35%)	33 (20%)	
White	311 (49%)	216 (46%)	95 (58%)	
Insurance				0.06
Private	320 (51%)	224 (48%)	96 (58%)	
Medicare/Medicaid	308 (49%)	240 (51%)	68 (41%)	
Other	6 (1%)	5 (1%)	1 (1%)	
Comorbidities				
Diabetes mellitus, yes	72 (11%)	61 (13%)	11 (7%)	0.02
Hypertension, yes	194 (31%)	149 (32%)	45 (27%)	0.16
Congestive heart failure, yes	16 (3%)	12 (3%)	4 (2%)	0.59
Coronary artery disease, yes	18 (3%)	10 (2%)	8 (5%)	0.07
Liver disease, yes	20 (3%)	15 (3%)	5 (3%)	0.58
Kidney disease, yes	115 (18%)	84 (18%)	31 (19%)	0.44
COPD, yes	30 (5%)	25 (5%)	5 (3%)	0.16
Asthma, yes	93 (15%)	73 (16%)	20 (12%)	0.17
Anemia, yes	4 (1%)	4 (1%)	0 (0%)	0.30
ASA class				0.11
1	62 (10%)	40 (8%)	22 (13%)	
2	395 (62%)	290 (62%)	105 (64%)	
3	172 (27%)	134 (29%)	38 (23%)	
4	5 (1%)	5 (1%)	0 (0%)	
Surgery				
Hysterectomy, yes	232 (37%)	197 (42%)	35 (21%)	<0.001
Sacrocolpopexy, yes	175 (28%)	143 (31%)	32 (19%)	0.004
Uterosacral ligament suspension, yes	48 (8%)	44 (9%)	4 (2%)	0.001
Sacrospinous ligament supension, yes	24 (4%)	20 (4%)	4 (2%)	0.21
Colpocleisis, yes	33 (5%)	25 (5%)	8 (5%)	0.50
Anterior repair, yes	199 (31%)	171 (37%)	28 (17%)	<0.001
Posterior repair, yes	248 (39%)	208 (44%)	40 (24%)	<0.001
Midurethral sling, yes	311 (49%)	231 (49%)	80 (49%)	0.47
Sacral nerve modulation, yes	11 (2%)	8 (2%)	3 (2%)	0.58
Cystoscopy, yes	428 (68%)	316 (67%)	112 (68%)	0.49
Hysteroscopy, D&C, yes	58 (9%)	47 (10%)	11 (7%)	0.13
Botox, yes	1 (0%)	1 (0%)	0 (0%)	0.74
Surgery type				<0.001
Major	394 (62%)	323 (69%)	71 (43%)	
Minor	240 (38%)	146 (31%)	94 (57%)	

*BMI* body mass index, *COPD* chronic obstructive pulmonary disease, *ASA* American Society of Anesthesiologists, *D&C* dilation and curettage

Table 1 also displays significant differences between women who had preoperative laboratory assessment and those who did not. Black women were more likely to have preoperative labs performed whereas no labs were associated with white race (*p* = 0.004). Women with diabetes mellitus were more likely to have preoperative laboratory assessment (*p* = 0.02). Women undergoing surgeries including hysterectomy (*p* < 0.001), sacrocolpopexy (*p* = 0.004), uterosacral ligament suspension (*p* = 0.001), anterior repair (*p* < 0.001), and posterior repair (*p* < 0.001) were more likely to have preoperative labs performed. Women undergoing major procedures were more likely to have preoperative labs completed (69% versus 43%, *p* < 0.001) than those undergoing minor procedures.

Four hundred and sixty-nine (74%) women had preoperative laboratory assessment prior to surgery compared with 165 (26%) who had no preoperative labs performed (Table 2). CBC was the most common lab collected (69%) followed by BMP or CMP (51%) and T&S (39%). Clinically meaningful laboratory abnormalities ranged from 0% to 6%. Hgb <10 mg/dl was the most common meaningful laboratory abnormality detected (6%), followed by Cr >1.0 mg/dl (3%), K <3.5 mEq/l (2%), and Na <136 mEq/dl (2%). All other detected laboratory abnormalities were < 1%. No cases were cancelled or postponed because of the detection of meaningful laboratory abnormalities preoperatively.

**Table 2:** Preoperative labs and clinically meaningful abnormalities

Variable	*N*, %
Preoperative labs	
T&S	250 (39)
CBC	437 (69)
BMP or CMP	320 (51)
No labs	165 (26)
Laboratory abnormality	
Hgb <10 mg/dl	29 (6)
Plt <100x10^3^/ml	2 (0)
Cr >1.0 mg/dl	21 (3)
Na <136 mEq/l	11 (2)
Na >145 mEq/l	0 (0)
K <3.5 mEq/l	12 (2)
K >5.1 mEq/l	4 (1)
Glucose >200 mg/l	1 (0)
Surgery cancelled, yes	0 (0)

*T&S* type and screen, *CBC* complete blood count, *BMP* basic metabolic panel, *CMP* complete metabolic panel

## Discussion

Three out of 4 women undergoing surgery for pelvic floor disorders had preoperative labs performed. Almost two-thirds of women had a CBC drawn and half of the study cohort had a T&S and BMP or CMP completed. Clinically meaningful laboratory abnormalities were rarely discovered prior to surgery. Despite detection of laboratory abnormalities, no preoperative adjustments were made to address the abnormalities, and no surgeries were cancelled or postponed. Our study reinforces the low utility of performing preoperative laboratory assessment in women undergoing surgery for pelvic floor disorders. Current hospital policies and procedures need to be re-evaluated to reflect current national guidelines of selective testing.

Current ASA guidelines for pre-anesthesia evaluation recommend against routine preoperative laboratory assessment [4]. At this institution, the primary care or anesthesia teams order these laboratory tests for medical clearance, and these clinicians are encouraged to evaluate patient characteristics and co-morbidities to determine specific indications for preoperative laboratory testing. Type and invasiveness of the procedure and specific medical co-morbidities (anemia, hematological disorder, renal disorder, liver dysfunction, etc.) can help to guide the decision to perform preoperative laboratory assessment that would provide valuable information to aid in determining the patient’s risk of a perioperative adverse event or guide anesthesia management [4, 8]. No explicit parameters for ordering preoperative tests have been identified. Instead, the Anesthesia Task Force recommends individualized testing based on specific clinical characteristics obtained from patient medical records, interviews, physical examination, and the type and invasiveness of the planned procedure [4]. Routine preoperative labs, defined by the absence of clinical indication or purpose [4], do not provide a significant contribution to a patient’s preoperative management and instead is associated with low patient satisfaction, increased laboratory costs, and inappropriate waste of laboratory resources and time [6, 7, 9, 10]. Women undergoing surgery for pelvic floor disorders are often healthy, with low rates of significant medical co-morbidities [3]. Surgical management of pelvic floor disorders are also considered low risk of perioperative adverse events or blood transfusions [1, 2]. Despite this, 3 out of 4 women underwent preoperative laboratory assessment in our study and clinically meaningful lab abnormalities were rarely discovered. Even if meaningful results were found, no preoperative interventions or surgical cancellations or postponements were made because of these abnormalities. Our findings are consistent with those of previous literature that highlights the low utility of routine preoperative testing within this patient population, emphasizing the need for updated hospital policies and practices [3, 5].

Prior literature has emphasized the limited clinical utility of routine preoperative laboratory assessment in women undergoing surgery for pelvic floor disorders [3, 5, 11, 12]. However, our study emphasizes that routine preoperative laboratory assessment continues to persist in surgical practice. Three-quarters of our cohort were undergoing at least one preoperative lab; however, nearly half had no significant medical co-morbidities and the most common comorbidity was hypertension. The cohort included women with a history of liver disease, kidney disease, and anemia, yet diabetes mellitus was the only co-morbidity that was associated with preoperative lab collection. Although preexisting diabetes may warrant presurgical blood tests, preoperative hemoglobin A1C is recommended to provide more useful insights into glucose control and risk of perioperative adverse events, not T&S, CBC, or CMP/BMP [8]. Metabolic panels can provide immediate blood glucose levels and acidosis status, but these findings are rarely helpful for surgical planning outside of poorly controlled diabetics [7, 8]. Invasiveness of the procedure was significantly associated with preoperative laboratory assessment, which is consistent with current national guidelines. Women undergoing major procedures were more likely to have preoperative labs performed than women undergoing minor procedures. However, when identifying specific surgeries, the outcomes were not consistent that women undergoing all major procedures were more likely to have preoperative laboratory testing. Although level of invasiveness is consistent with current preoperative test guidelines, major surgery categorization alone is not sufficient to warrant preoperative laboratory assessments.

Hysterectomies are commonly performed at the time of reconstructive pelvic surgery for pelvic organ prolapse. Prior to the implementation of minimally invasive techniques, open hysterectomy was associated with greater blood loss and a higher risk of perioperative blood transfusion [1, 2]. Perioperative transfusion risk for women undergoing surgery for pelvic floor disorders is less than 1% and previous studies have found that an abdominal approach was associated with a 5.5 times higher odds of receiving a perioperative blood transfusion compared with vaginal approach [2]. The shift toward minimally invasive surgical techniques in women undergoing major urogynecological procedures (i.e., hysterectomy, apical suspension) has significantly reduced perioperative transfusion risk. Therefore, we argue that even patients undergoing “major” surgical procedures for pelvic floor disorders do not qualify for preoperative laboratory assessment without other patient factors that increase the risk of perioperative adverse events.

Despite current national guidelines, preoperative laboratory assessment is often performed frequently without indication and present limited utility. Previously, literature has determined patient risk factors that should guide pre-anesthesia evaluation and testing. Our findings identify an opportunity for hospitals to improve the current standard of care and reflect these guidelines. Further research is also needed to identify if adherence to these national guidelines decreases unnecessary preoperative testing and health care costs. Studies are also needed to identify if patient satisfaction or preoperative testing utility improves when following current ASA recommendations.

Our study has several strengths and limitations. We found that factors associated with preoperative labs did not reflect current national guidelines outside of invasiveness of the surgical procedure. This study adds to the growing bulk of literature demonstrating the high proportion of preoperative labs performed without indication and the low utility of such labs when abnormal values are identified. This study presents an opportunity to improve the practice patterns at our institution to better reflect national guidelines. Our study was performed at an urban, high-volume tertiary care center and included a large study cohort. Although we recognize that this also limits the generalizability of our findings when compared with studies utilizing large national databases, these databases do not provide all clinically meaningful laboratory abnormalities, which we were able to assess during review of electronic medical records. Databases also do not provide details regarding preoperative intervention or surgical cancellation or postponement, which we were able to abstract from the patient chart as well. Limitations include the retrospective nature and potential for information bias inherent to the study design. This study highlighted surgical postponement and cancellation; however, we did not account for intraoperative and postoperative management based on laboratory findings. Because the electronic medical records lack clear documentation describing the indication for each lab obtained, we could not discern which tests were ordered with comorbidity indication or routinely without indication. We were also limited to the electronic medical record at our own institution. Patients may have had preoperative labs performed by outside providers that were not immediately available during our review. There may also have been missing data or coding errors that we could not control for. Future directions for this study include narrowing the cohort to exclude patients with comorbidities that indicate preoperative testing to evaluate difference in routine testing.

Routine preoperative labs are discouraged owing to the financial and logistical burdens that often provide no clinical significance for women undergoing surgery for pelvic floor disorders. Instead, utilizing individualized risk stratification can identify the patients who may benefit from preoperative labs. However, within our cohort, we found a high proportion of surgical patients who underwent at least one preoperative test, with minimal lab abnormalities identified and no case cancellations, preoperative adjustments, or postponements. Hospital policies and regulations are lagging behind national guidelines for pre-anesthetic assessment to determine the risk of adverse perioperative outcomes and their influence on surgical planning or cancellations. Future directions include implementing quality improvement initiatives to enhance patient care and decrease unnecessary testing and health care costs.

## Data Availability

Data is not available for external use.
